# Insights into the Origin of Distinct Medin Fibril Morphologies Induced by Incubation Conditions and Seeding

**DOI:** 10.3390/ijms19051357

**Published:** 2018-05-03

**Authors:** Hannah A. Davies, Chiu Fan Lee, Leanne Miller, Lu-Ning Liu, Jillian Madine

**Affiliations:** 1 Institute of Integrative Biology, University of Liverpool, Liverpool L69 7ZB, UK; h.davies1@liverpool.ac.uk (H.A.D.); lmiller4@liv.ac.uk (L.M.); luning.liu@liverpool.ac.uk (L.-N.L.); 2 Department of Bioengineering, Imperial College London, London SW7 2AZ, UK; c.lee@imperial.ac.uk; 3 Department of Physics, University of Liverpool, Liverpool L69 7ZE, UK

**Keywords:** atomic force microscopy, amyloid, aortic medial amyloid/medin, mathematical modelling, aggregation

## Abstract

Incubation conditions are an important factor to consider when studying protein aggregation in vitro. Here, we employed biophysical methods and atomic force microscopy to show that agitation dramatically alters the morphology of medin, an amyloid protein deposited in the aorta. Agitation reduces the lag time for fibrillation by ~18-fold, suggesting that the rate of fibril formation plays a key role in directing the protein packing arrangement within fibrils. Utilising preformed sonicated fibrils as seeds, we probed the role of seeding on medin fibrillation and revealed three distinct fibril morphologies, with biophysical modelling explaining the salient features of experimental observations. We showed that nucleation pathways to distinct fibril morphologies may be switched on and off depending on the properties of the seeding fibrils and growth conditions. These findings may impact on the development of amyloid-based biomaterials and enhance understanding of seeding as a pathological mechanism.

## 1. Introduction

Amyloid formation proceeds via a nucleation-dependent mechanism, during which monomers and multimers assemble to form a critical nucleus, followed by rapid elongation into insoluble fibrils [1]. A number of factors are known to affect nucleation, including: cofactors/small molecules, metal ions, pH, ionic strength, and agitation [2,3,4,5,6]. Agitation has been shown to reduce the lag time for nucleation of amyloid formation in vitro for amyloidogenic proteins including amyloid-β [7,8], β_2_-microglobulin [9], insulin [10] and amylin [11]. In addition to enhancing fibrillation kinetics, different amyloid-β fibril morphologies can be induced, dependent upon agitation conditions in vitro [12].

Amyloid fibrillation, like many polymerisation reactions, can be accelerated through the addition of a preformed template species to eliminate the lag phase [13]. Seeding usually requires a high degree of similarity between the seed and the elongating species (i.e., same protein) but there is evidence that cross-seeding can occur between different proteins [14]. In addition to sequential similarity, it has been shown that conformational differences can also affect seeding efficiency. Proteins that share the same sequential information but adopt different conformations are known as strains in the context of self-assembly/aggregation [15].

The identification of functional amyloid-like fibrils in nature has enhanced the possibilities of using engineered amyloids as functional nanomaterials. Bacteria [16], algae [17] and fungi exploit the properties of amyloid-like fibres, specifically the ability to switch between soluble and fibrillar forms for matrix generation enabling interactions, adhesion and epigenetic information storage, respectively. In addition to pathological amyloid in humans, amyloid assembly has also been identified to form a scaffold as a template to orient and accelerate fibril growth during melanin synthesis and avoid toxic intermediate conformations [18], and for the stabilisation of peptide hormones in secretory granules [19].

The growth rate and rate of fragmentation are key parameters that alter the biomechanical properties of the resultant fibrils. The ability to manipulate the fibril morphology and, in turn, functional properties is an attractive feature for the use of amyloid-like fibrils in biotechnology. Therefore, understanding the underlying mechanism controlling fibril growth is essential to determine and manipulate conditions to produce a homogenous and reproducible material. We set out here to probe the effects of agitation on medin—an amyloid protein deposited in the aorta—and to investigate the seeding potential of fibrils with different morphologies (strains). This was studied using biophysical techniques: thioflavin T (ThT), circular dichroism (CD) and intrinsic fluorescence (IF), nuclear magnetic resonance (NMR) and atomic force microscopy (AFM). Subsequently, we employed biophysical modelling to provide a qualitative explanation of the experimental observations. We showed that agitation has a dramatic effect on the fibrillation properties of medin, producing fibrils with distinctive morphologies, which, as seeds, in turn produce further different morphologies. Understanding of the kinetics of aggregation and fibril structure of medin may have important roles in elucidating the molecular mechanisms of aggregation in vivo and help understanding of amyloid polymorphism and the role of fragmentation in biomaterial development.

## 2. Results

**Figure 1:**
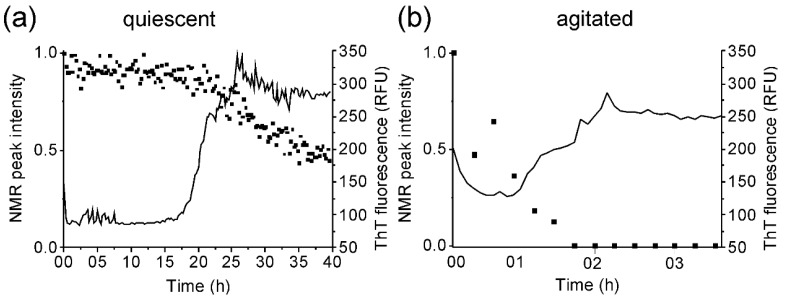
Fibrillation of medin. Thioflavin (ThT) fluorescence (solid line) for 20 µM medin incubated in 20 mM NaPhos, 20 mM NaCl, pH 7.4 at 37 °C under (**a**) quiescent and (**b**) agitated conditions. Results are shown as the mean of three replicates. NMR peak intensity (symbols) normalised to starting intensity.

Fibrillation of medin was assessed by ThT fluorescence. Quiescent incubation of medin showed a lag time prior to fibrillation of approx. 18 h, followed by an increase in ThT fluorescence (Figure 1a, solid line). In contrast, agitation enhanced aggregation with a rapid growth stage after approx. 60 min (Figure 1b, solid line). NMR peak intensity for all residues showed a loss of intensity starting at approx. 18 h for quiescent incubation, consistent with an increase in size of aggregate species (Figure 1a, symbols). Incubation with agitation resulted in a reduction in intensity after 15 min with complete intensity loss observed after 1.5 h (Figure 1b, symbols). NMR “invisible states” result when exchange rates between species are on the same timescale as the NMR experiment is collected (i.e., ms) resulting in averaged peaks which cannot be observed. Alternatively, an increase in correlation time, which is related to the tumbling rate in solution (and, in turn, the size and morphology), would result in loss of intensity. The differences in NMR peak intensity at the end of incubation between quiescent (0.5) and agitated incubation (0) (Figure 1) therefore suggested differences in final morphologies and/or exchange rates between species in solution dependent upon incubation conditions. It is also noted that for agitated incubation, NMR intensity is lost before ThT fluorescence is observed, suggesting the formation of an early on-pathway intermediate that does not bind ThT [20].

**Figure 2:**
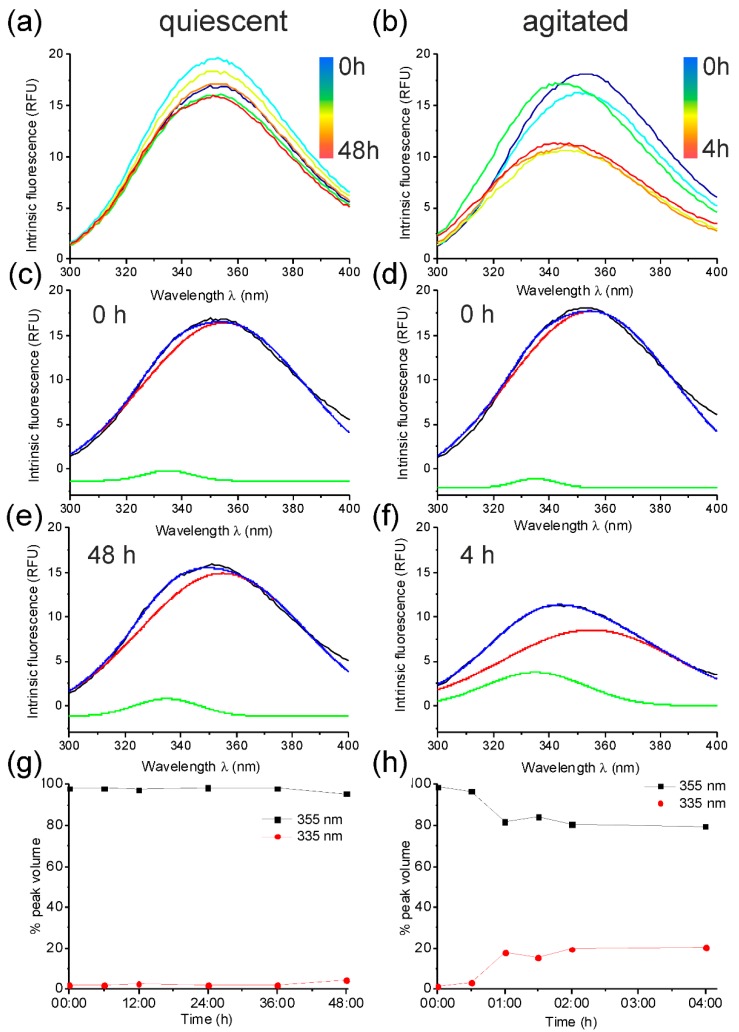
Intrinsic fluorescence of medin. Tryptophan fluorescence emission for 20 µM medin incubated in 20 mM NaPhos, 20 mM NaCl, pH 7.4 at 37 °C under quiescent (left) and agitated conditions (right). (**a**,**b**) Spectra were measured at intermediate timepoints during aggregation (0, 6, 12, 24, 36 and 48 h for quiescent and 0, 0.5, 1, 1.5, 2 and 4 h for agitated). Fluorescence spectra (black) for start (0 h, (**c**,**d**)) and end (48 h, (**e**) and 4 h, (**f**)) were deconvoluted with nonlinear least-squares fitted spectra (blue) approximated by two Gaussian components centred at 355 nm (red) and 335 nm (green) (see Methods for full details). (**g**,**h**) The areas of the Gaussian curve fits (centred at 355 and 335 nm as shown in (**c**–**f**)) were used to calculate the fraction of each contributing to the overall spectra at each timepoint.

Medin contains two tryptophan residues at positions 11 and 21. Intrinsic tryptophan fluorescence investigates the relative solvent exposure of these residues [21]. The IF spectrum for freshly prepared, soluble medin had an emission maximum at 355 nm, suggesting that the tryptophan residues were solvent exposed (i.e., unfolded) (Figure 2). This is consistent with previous CD data suggesting medin contains a mixture of random coil and β-sheet structures [22,23,24]. Upon incubation, this peak displayed a blue shift (towards a lower wavelength), suggesting that the tryptophan residues were becoming less solvent exposed (i.e., buried within a secondary structure conformation) (Figure 2a,b). Deconvolution of the IF spectra for medin aggregation under agitated and quiescent conditions with nonlinear least-squares fitted spectra approximated by two Gaussian components—centred at 355 nm (solvent-exposed) and 335 nm (buried)—gave different profiles for aggregation over time, dependent upon incubation conditions (Figure 2g,h). Quiescent conditions showed a minimal shift in peak maxima (Figure 2c,e), which was reflected in the deconvolution summary (Figure 2g). However, agitated growth gave a larger shift of up to 10 nm (Figure 2d,f), and a larger change in volume upon deconvolution, which occurred at approx. 60 min, consistent with the lag time observed by ThT fluorescence (Figure 1b and Figure 2h). Agitated growth results in fibrils, in which tryptophan residues are less exposed to the surrounding solvent than in fibrils grown under quiescent conditions.

**Figure 3:**
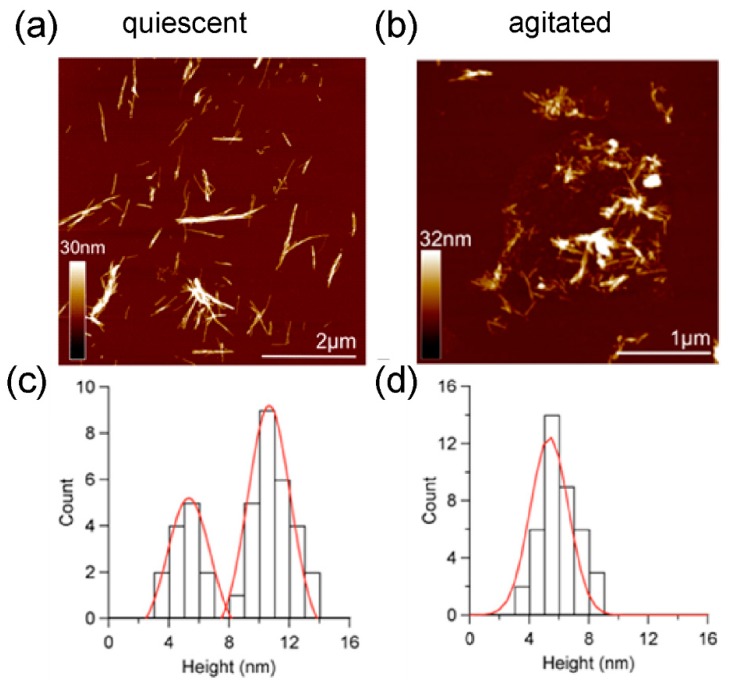
Atomic Force Microscopy Analysis. Fibrils formed under (**a**) quiescent and (**b**) agitated conditions. Height distribution analysis shows (**c**) two height populations of 5.1 ± 1.0 (*n* = 170) and 10.7 ±1.4 nm (*n* = 90) for quiescent fibrils, (**d**) with only one species of average height 5.3 ± 1.1 nm for agitated fibrils.

AFM analysis showed that quiescent incubation resulted in fibrils with two distinct height populations of 5.1 ± 1.0 nm and 10.7 ± 1.4 nm (Figure 3a,c) with observable periodicity of 22–198 nm (see [24]). When incubated with agitation, a denser, more homogeneous population of fibrils was produced (Figure 3b), with height 5.3 ± 1.1 nm (Figure 3d), and no observable periodicity. These data are consistent with observations for amyloid-β, where agitation gives straight rods, approx. 6 nm in width [12]. In contrast, quiescent growth gave amyloid-β fibrils with a modulation in width, which can be propagated through successive generations of seeding to give uniform fibrils with periodicity modulation of 120 ± 20 nm [4]. Previous structural analysis of monomeric medin suggests core β-strands of 2–2.5 nm, suggesting that 5 nm fibrils observed here may assemble from two layers of sheets [25].

**Figure 4:**
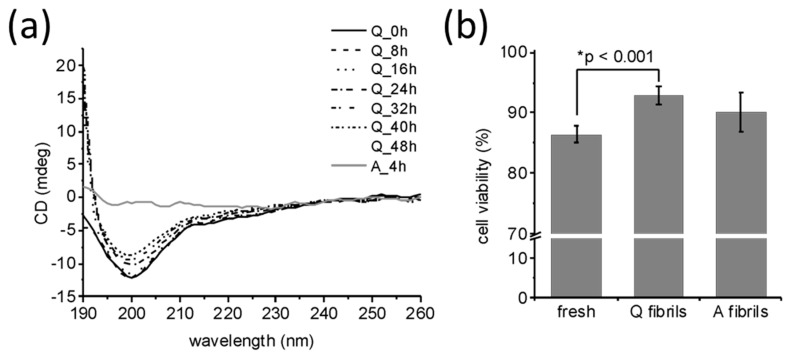
Properties of fibrils formed under quiescent and agitated incubation. (**a**) Circular dichroism (CD) shows that following incubation of medin for 48 h under quiescent (Q) conditions there is still a CD signal remaining (dashed line), whereas incubation under agitated (A) conditions has no detectable CD signal remaining (dotted lines). Analysis of the spectra using CONTINLL suggests that quiescent fibrils have increased β-sheet structures compared to freshly prepared medin (Table 1). (**b**) Cell viability of human aortic smooth muscle cells (HAoSMC) after exposure to freshly prepared and aggregated medin, as determined by Cell Counting Kit-8 (CCK-8) assay; mean ± SEM is shown for *n* = 5. There is no statistical difference between A and Q fibrils assessed by *t*-test.

**Figure 5:**
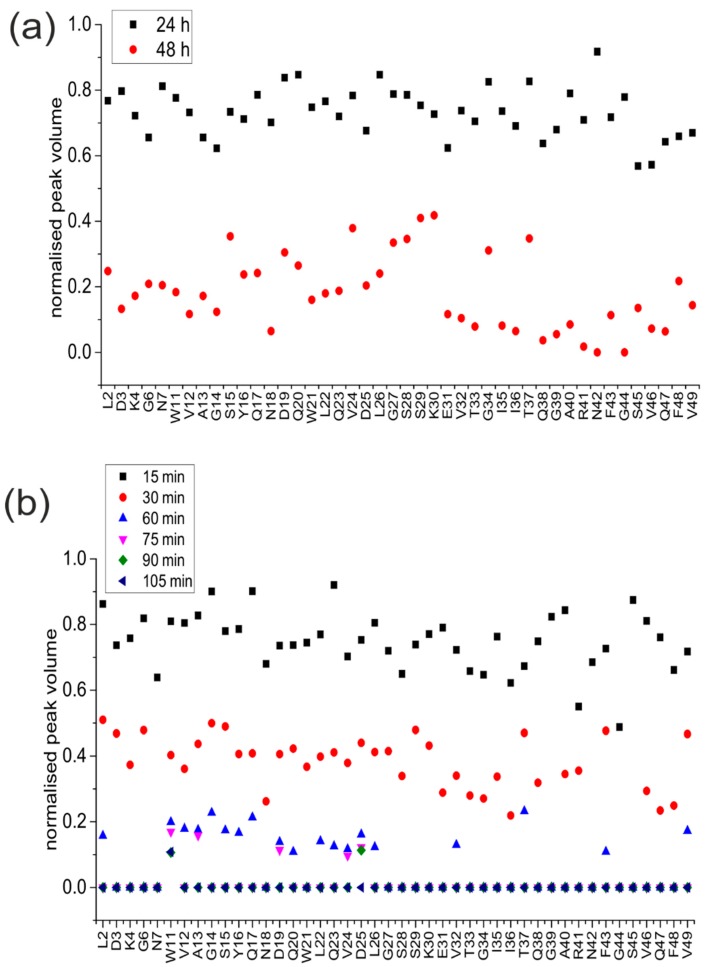
Site-specific NMR analysis. Normalised peak volume relative to starting volume for (**a**) quiescent incubation at 24 and 48 h, and (**b**) agitated incubation at 15, 30, 60, 75, 90 and 105 min.

Circular dichroism was used to analyse differences in secondary structure of medin following incubation (Figure 4a). Analysis showed that freshly prepared medin was largely unordered with approximately 30% β-sheet, consistent with previous observations [22,23,24]. Upon incubation under quiescent conditions, there was a change in CD spectra indicating enhanced β-sheet structure and reduced unordered and helix (Figure 4a and Table 1). However, upon incubation with agitation, there was no observable CD signal (Figure 4a, grey line). This is consistent with NMR data presented in Figure 1a showing that following incubation under quiescent conditions there was still approximately 50% intensity remaining, suggesting that soluble species were still present within the solution. In contrast, upon incubation with agitation, NMR and CD signal intensity were completely lost (Figure 1b and Figure 4a), suggesting that a larger percentage of medin has been converted into insoluble fibrillar species. IF also showed a larger decrease in intensity when incubated under agitated conditions compared to quiescent (Figure 2), consistent with less soluble species remaining upon agitated incubation. Site-specific analysis of NMR data revealed that signals from the central region (residues 15–30 for quiescent incubation and 11–26 for agitated incubation) remain visible for longer than N- and C-terminal regions (Figure 5). Selective peak disappearance suggests small oligomer formation [26], with N- and C-terminal regions driving the self-association process, consistent with the mechanism proposed previously by molecular modelling [25]. Upon agitated incubation, peaks disappear from residue 27 onwards by 60 min, compared with greater loss from residue 31 onwards upon quiescent incubation (Figure 5), suggesting different assembly mechanisms may occur at this initial point of self-association and oligomer formation.

Toxicity analysis using human aortic smooth muscle cells (HAoSMC) showed that freshly prepared medin displayed toxicity of approximately 15% (Figure 4b). There were no significant differences between the two fibril forms, both showing approximately 10% toxicity compared to live buffer control. This data is consistent with previous observations that medin fibrils are less toxic than freshly prepared [23,24].

**Figure 6:**
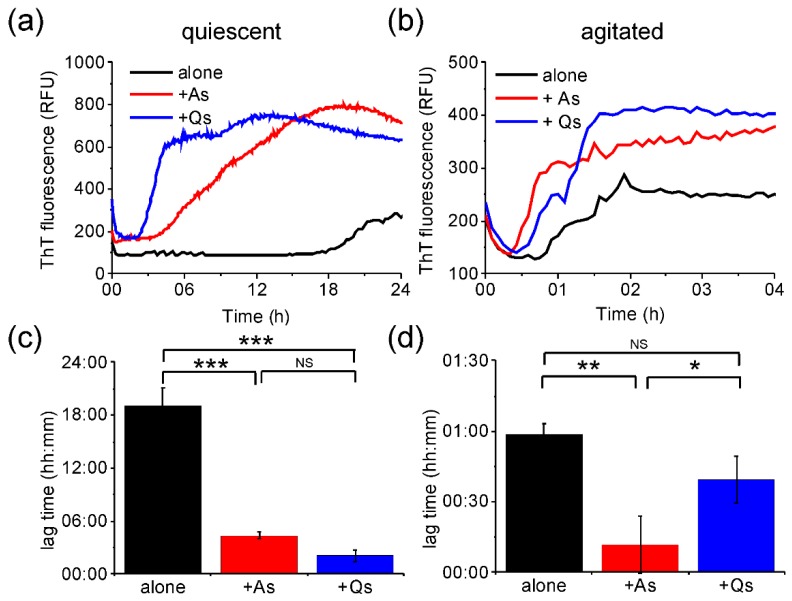
Fibrillation of medin in the presence of pre-formed medin seeds. ThT fluorescence for 20 µM medin incubated in 20 mM NaPhos, 20 mM NaCl, pH 7.4 at 37 °C under (**a**) quiescent (**b**) and agitated conditions. Medin alone (black), and in the presence of 5% *v*/*v* preformed fibril seeds formed with agitation (As, red), or quiescent (Qs, blue). Results are shown as the mean of three replicates. Lag time calculated by fitting the ThT curves as described previously [23,27] for (**c**) quiescent and (**d**) agitated data. Data are shown as the mean ± SD for triplicate curves. NS non-significant; * *p* < 0.05; ** *p* < 0.005; *** *p* < 0.00005. *p* values were obtained using ANOVA with Bonferroni means comparison.

The ability of preformed fibrils produced under quiescent and agitated conditions to act as seeds to promote fibrillation of soluble medin was also assessed by ThT fluorescence. Preformed fibril strains prepared under agitated (A) or quiescent (Q) conditions were sonicated to produce seeds [12] and added to soluble medin to 5% *v*/*v* at the start of the incubation and ThT data collection. AFM analysis of the sonicated fibrils used as seeds showed that, following sonication of preformed quiescent fibrils, only one height population of 4.9 ± 1.5 nm was present (Figure S1). This suggests that the larger fibril species observed for quiescent fibrils prior to sonication are more susceptible to the breakage effects of sonication. Under both conditions (quiescent and agitated), addition of seeds reduced the lag time required for fibrillation (Figure 6). Under the quiescent, slow aggregation conditions, a significant reduction in lag time was observed for the addition of both Q and A seeds, of 17 and 14.5 h, respectively (Figure 6a,c). In contrast, under agitated conditions, only the addition of A seeds led to a significant reduction in lag time of 50 mins (Figure 6b,d). It is interesting to note that seeds formed under the same conditions as the test conditions were more efficient at enhancing nucleation. This suggests that there are differences between the properties of the seeds that alter their efficiency for reducing lag time. AFM analysis of the seeds (Figure S1) suggests that the seeds are of similar height (~5 nm), therefore, this alone cannot explain this observation. It is also noted that the final fluorescence was greater in the presence of seeds than aggregation alone for both incubation conditions (Figure 6), suggesting that seeding increased the fibril yield or altered the fibril morphology in addition to altering nucleation lag time. The increase in fluorescence could also indicate that addition of seeds caused a shift from an off-pathway intermediate formed during the first incubation to enhanced fibre production in the presence of seeds. This is particularly evident for quiescent conditions where NMR and CD analyses show soluble species remaining (Figure 1a and Figure 4a), which could represent an off-pathway intermediate state.

**Figure 7:**
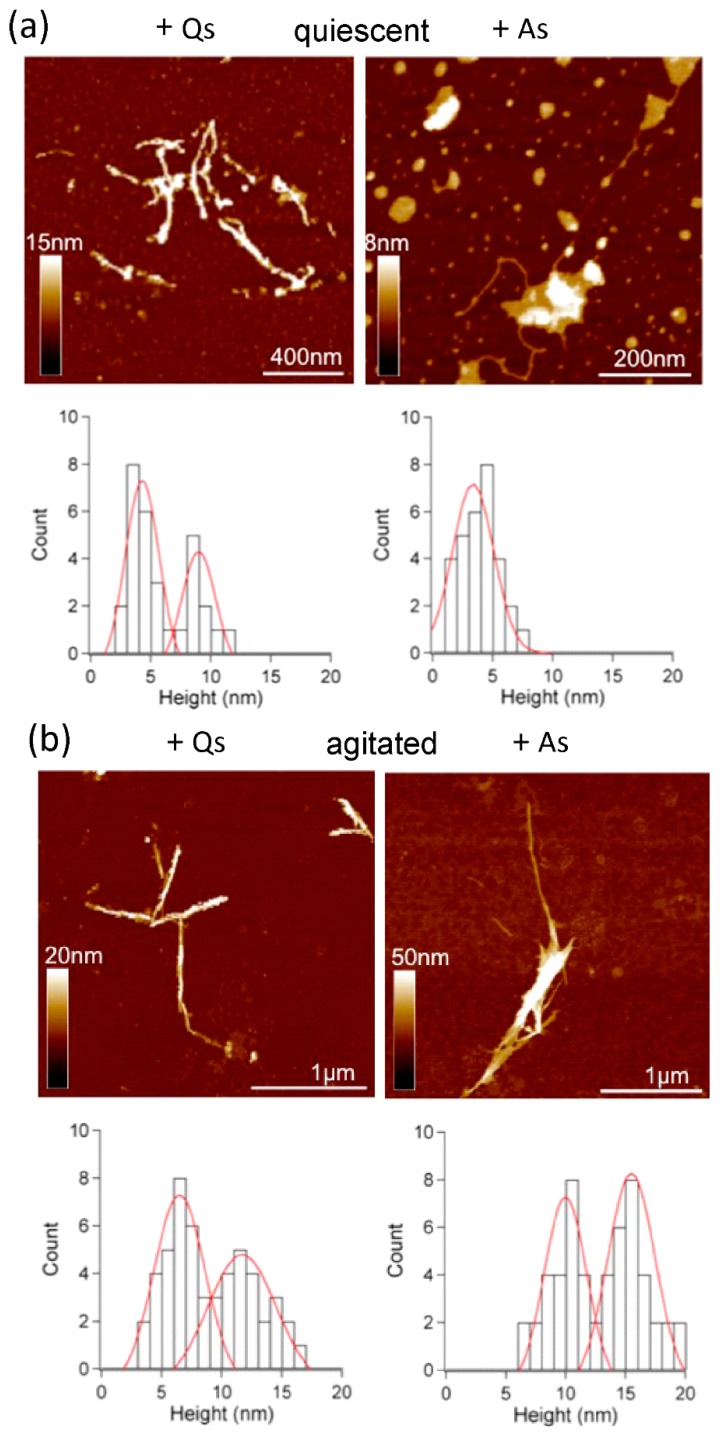
Atomic Force Microscopy Analysis. Fibrils formed under (**a**) quiescent and (**b**) agitated conditions with the addition of 5% *v*/*v* seeds preformed with quiescent (Qs) or agitated (As) incubation. Height distribution analysis for quiescent incubation shows height populations of 4.2 ± 1.2 nm and 9.1 ± 1.2 nm (*n* = 40) with addition of A seeds (**a**, left), and 3.5 ± 1.3 nm (*n* = 40) with addition of Q seeds (**a**, right). Height distribution analysis for agitated incubation shows height populations of 6.4 ± 1.5 nm (*n* = 40) with addition of Q seeds (**b**, left), and 9.9 ± 2.5 nm (*n* = 40) with addition of A seeds (**b**, right).

**Figure 8:**
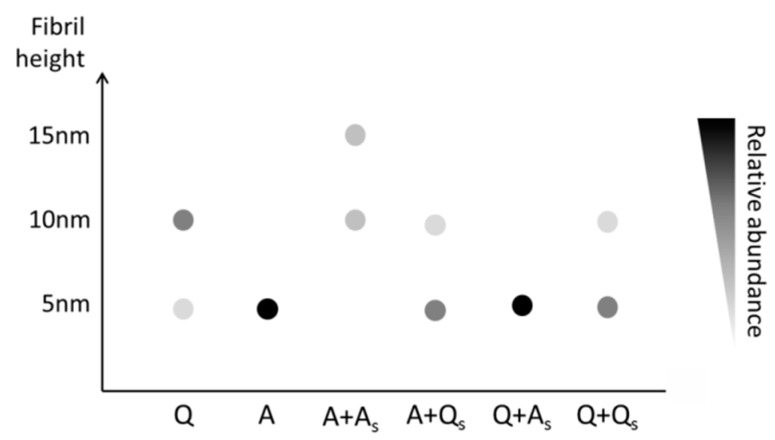
Schematic of the height measurements by AFM performed on fibrils prepared under different conditions. Quiescent (Q) incubation generates two species of fibrils with heights 5 and 10 nm, whereas agitated (A) growth only forms 5 nm fibrils. Upon seeding with seeds formed from sonicated A fibrils (A_s_) or quiescent fibrils (Q_s_), fibril dimensions are largely determined by the seed species, except for A + A_s_ (see text for explanation of this phenomenon).

AFM analysis of seeded medin fibrils showed two populations for most conditions, with the exception of the addition of A seeds during quiescent incubation (Figure 7a, right). Height analysis of all AFM data showed three main populations with heights approx. 5, 10 and 15 nm (Figure 6 and Figure 7). A summary of these results is presented as a schematic in Figure 8.

Based on the AFM height measurement alone, three types ($s_{1},\;s_{2},s_{3}$) of fibrils can be identified, which were around 5, 10 and 15 nm, respectively (Figure 8). Focusing first on the system without sonicated seeds (the first two columns in Figure 8), we observe that, under the quiescent condition (Q), both $s_{1}$ and $s_{2}$ can be nucleated, while under the agitated condition (A), species $s_{1}$ dominates. When these incubated seeds are sonicated and then used to seed the system under both quiescent and agitated conditions, we see that the species present in the system remains the same, except for the case of the system under agitated condition seeded by sonicated fibrils incubated under agitated condition (A + A_s_). In this case, species $s_{1}$ seems to disappear from the system, while species $s_{2}$ and $s_{3}$ emerge.

To rationalise the emergence of such a species, we assume that the thicker fibrils $\left(s_{2},s_{3}\right)$ originate from type $s_{1}$. Specifically, we assume that fibrils of type $s_{2}$ consist of the braiding of *two* fibrils of type $s_{1}$, and fibrils of type $s_{3}$ consist of the braiding of three fibrils of type $s_{1}$. We further assume that the likelihood for two fibrils of type $s_{1}$ to come together to form a fibril of type $s_{2}$ decreases with the fibril length. In other words, our assumption was based on the intuition that it is easier to “braid” a rope out of short fragments of filaments than to do so with long filaments. With these assumptions in mind, we can explain why fibril of type $s_{3}$ emerges under the (A + A_s_) conditions. We know that the fibrils incubated under agitated condition is of type $s_{1}$, after sonication, these fibrils are fragmented and we thus have an abundance of short fibrils of type $s_{1}$. When these are used to seed a system under agitated condition, both fibrils of types $s_{2}$ and $s_{3}$ can be nucleated from these seeds and, indeed, exhaust the original $s_{1}$ seeds to the extent that the abundance of fibrils of type $s_{1}$ become negligible. In contrast, when the system is initiated without seeding and incubated under the agitated condition, short fibrils of type $s_{1}$ are nucleated, but then due to the fast elongation rate, fibrils of type $s_{1}$ in their short form only exist transiently before elongation effectively eliminates their ability to come together to nucleate other types of thicker fibrils.

To complement the physical picture presented in the discussion above, we have used mathematical modelling to verify the plausibility of the above scenario in the Supplementary Information (SI). We note that given the inherent complexity in the system and the diverse physical processes (diverse nucleation pathways, distinct elongation rates for different types of fibrils, etc.), our mathematical model in the SI aims only to demonstrate that the above physical picture can indeed replicate the experimental observation.

## 3. Discussion

Incubation conditions can alter amyloid aggregation characteristics, and agitation can dramatically alter fibril morphologies [4]. Here, we present three distinct morphologies and mechanisms of aggregation for the amyloidogenic protein medin, dependent upon in vitro incubation conditions. These data serve as a reminder that in vitro analysis of factors influencing aggregation for potential pathological investigations or biomaterials development needs careful consideration of incubation conditions [28]. Data presented here suggested that the final morphology of fibrils produced in the presence of seeds formed by sonication of preformed fibrils was dictated by the morphology of the seed species. However, there was one exception to this finding: the addition of agitated seeds during agitated incubation (Figure 7 and Figure 8). By using biophysical modelling presented in the Supplementary Information, we are capable of rationalising the evolution of distinct fibril morphologies based on the seeding and incubation conditions. In this model, nucleation to the narrowest fibrils occurs from the monomeric pool. Wider fibrils are subsequently formed by lateral association of the narrowest fibrils. This is consistent with periodicity occurring only in quiescent 10 nm fibrils that have formed from two 5 nm fibrils winding together. Specifically, modelling results suggest that agitation can be a way of accessing a distinct pathway to fibril morphology that is otherwise inaccessible (generation of 15 nm fibrils). The modelling results (Figure S3) are capable of explaining the salient features of the experimental observations depicted in Figure 8. We emphasise here that the modelling results are aimed at providing a qualitative explanation of the experimental observations, instead of a quantitative fit to the experimental data.

Nucleation-dependent polymerisation and amyloid growth is concentration and time-dependent, however, air–water interfaces and agitation can overcome the critical concentration, enabling aggregation at a lower concentration with reduced nucleation time [29,30]. Agitation enhances nucleation, possibly due to fragmentation causing an increase in fibril ends available for addition of monomeric protein [31]. In vitro agitation could be representative of the conditions experienced in vivo due to convection of extracellular fluids in the body, as previously described for the brain [32]. Similarly, the aorta could provide a highly mobile environment under high shear forces and flow, which would influence the aggregation of medin in vivo.

Seeding is thought to be a key mechanism by which amyloid in disease spreads between cells and in tissues and from one organ to another [14]. In disease, different strains are responsible for much of the variation in pathology and disease transmission [33]. It has been noted that medin amyloid could influence the tissue distribution of serum amyloid A derived amyloidosis, with in vitro studies showing that medin amyloid-like fibrils could promote the fibrillation of serum amyloid A [34]. This study suggested that highly prevalent medin amyloid may initiate fibril formation of the more uncommon amyloidoses by a cross-seeding mechanism.

In addition to enhancing understanding of the implication of amyloid formation in disease pathologies, in vitro formation of a highly homogeneous population of fibrils, as shown here for agitated fibrils, has the potential for use in biomaterial design applications exploiting the mechanical properties of amyloid fibres [35]. We show here that we can manipulate the system using a combination of agitation and seeding to access a distinct pathway for fibril generation that was previously inaccessible. Understanding the process of hierarchical fibril assembly will enhance and expand the commercial application of amyloid-like materials.

## 4. Materials and Methods

### 4.1. Expression and Purification of Medin

Medin was expressed as previously described [24,36] using Lemo 21 (DE3) cells. Briefly, cell pellets were resuspended in 6 M guanidine hydrochloride (GdmCl), 20 mM sodium phosphate, 20 mM NaCl, pH 8 and frozen at −20 °C. Cells were homogenised, and cell debris removed by centrifugation (19,000× *g*, 15 min, 4 °C). The supernatant was loaded onto a 5 ml Ni^2+^-NTA column and washed with 4 column volumes (CV) of 6 M GdmCl, pH 8, followed by 4 CV of 6 M GdmCl, pH 6, and eluted with 3 CV of 6 M GdmCl, pH 2, and stored at −20 °C. Fusion protein was buffer exchanged into 20 mM sodium phosphate, 0.5 M NaCl, 20 mM imidazole, pH 7.4, and the His6-SUMO tag was removed by incubation with SUMO protease I at 4 °C for 3 h. The cleavage mixture was then passed through a 5 ml Ni^2+^-NTA column and the flow through containing medin was collected and characterised by matrix-assisted laser desorption and ionisation mass spectrometry.

### 4.2. Incubation of Medin

Medin was buffer exchanged into aggregation buffer (20 mM sodium phosphate, 20 mM NaCl, pH 7.4) and incubated at 20 µM at 37 °C under agitated (in 1.5 mL Eppendorf tubes containing a 2 × 2 mm magnetic flea) or quiescent conditions. For seeding experiments, preformed fibrils (20 µM) were sonicated (2 min, 10% duty cycle) and added to monomeric medin at 5% *v*/*v* [12].

### 4.3. Thioflavin T Fluorescence

ThT fluorescence assays were carried out on a Flexstation 3 microplate reader (Molecular Devices Ltd., San Jose, CA, USA). Experiments were carried out in 96-well, black-walled, clear-bottomed microplates (Nunc). Data were recorded every 5 min using bottom read mode, with excitation at 440 nm and emission at 490 nm. The assay was carried out using 20 µM medin with 2 µM ThT at 37 °C under two different conditions: agitated (with 250 s shaking in between each reading) and quiescent (5 s shaking before each reading). Shaking was linear, cycling 19.7 Hz and 29.41 Hz for 204 ms each.

### 4.4. NMR

Two-dimensional band-selective optimised flip-angle short-transient heteronuclear multiple quantum coherence (SOFAST HMQC) NMR spectra were acquired at 37 °C for 20 µM ^15^N medin containing 10% (*v*/*v*) ^2^H_2_O on a Bruker AVANCE III 600 MHz equipped with a 5 mm cryoprobe. Spectra were collected every 15 min and processed using Topspin 3.1 (Bruker, Billerica, MA, USA) with assignment carried out in CCPN Analysis [37] using chemical shift values from BMRB deposition 26,576 [38]. One-dimensional planes were processed from the 2D experiments collected. All 1D planes were integrated between 6.0 and 9.0 ppm in the direct dimension and normalised to the first experiment using Topsin 3.1. Agitated samples were incubated in Eppendorfs (as described above) as aliquots and transferred to 5 mm NMR tubes at the required timepoint.

### 4.5. Intrinsic Fluorescence

IF measurements were carried out on a Cary Eclipse Varian fluorescence spectrometer using 20 µM medin. Tryptophan residues were excited at 279 nm and the emission spectra recorded between 300 and 400 nm with a bandpass of 5 nm [21].

### 4.6. Atomic Force Microscopy

After incubation at 20 µM at 37 °C for 48 h, samples were incubated in 40 μL absorption buffer (10 mM Tris-HCl pH 7.4, 150 mM KCl, 25 mM MgCl_2_) [39] on freshly cleaved mica for 15 min, and then rinsed with sterile-filtered deionized water before drying. Samples were imaged in PeakForce QNM Mode in air on a Bruker Multimode 8 Atomic Force Microscope (AFM) equipped with a 160 μm scanner (J-scanner) using SCANASYST-AIR probes (*k* = 0.4 N/m). Images were recorded at 512 × 512 pixels and the AFM data were analysed using NanoScope and Gwyddion programs.

### 4.7. Cell Viability

Primary human aortic smooth muscle cells (HAoSMCs; Promocell, Heidelberg, Germany) were plated on 96-well plates at 4000 cells/well and grown for 24 h. Medin samples (control and incubated under quiescent or agitated conditions at 20 µM) were added to the cells to give a final concentration of 4 µM. After incubation for 48 h, 10 μL of Cell Counting Kit-8 (CCK-8) solution was added and further incubated for 2 h. Absorbance was then measured at 450 nm. Percentage cell viability was calculated based on the absorbance measured relative to that of cells exposed to buffer alone.

### 4.8. Circular Dichroism

Synchrotron radiation circular dichroism (SRCD) spectra were acquired for 50 μM medin in a 0.2 mm pathlength quartz cuvette from 190 nm to 260 nm on beamline B23 of the Diamond Light Source (Oxford, UK), in 1 nm increments using a 0.5 mm slit width. Spectra were recorded as the average of four scans and are presented after subtraction of buffer control spectra. Data were fitted using CONTINLL with basis set 8 in Olis GlobalWorks [40]. The percentages of α-helix, β-sheet, turn, and random coil content were recorded, S.D. values were 0.08 or below.

## Figures and Tables

**Table 1:** Secondary structure analysis of CD data shown in Figure 4. Data were fitted using CONTINLL with basis set 8 in Olis GlobalWorks. The percentages of α-helix, β-sheet, turn, and random coil content are shown.

Secondary Structure	Time (h)						
	0	8	16	24	32	40	48
**Helix**	18	23	0	0	0	0	0
**Sheet**	8	7	58	59	60	63	67
**Turn**	27	27	20	19	18	19	7
**Unordered**	47	42	22	21	22	18	25

## Supplementary Materials

Supplementary materials can be found at http://www.mdpi.com/1422-0067/19/5/1357/s1.

## Abbreviations

AFM	Atomic Force Microscopy
CCK-8	Cell Counting Kit-8
CD	Circular Dichroism
GdmC	Guanidine hydrochloride
IF	Intrinsic Fluorescence
NMR	Nuclear Magnetic Resonance
ThT	Thioflavin T
