# Supplementary material for: Insights into the Origin of Distinct Medin Fibril Morphologies Induced by Incubation Conditions and Seeding
Source: Int J Mol Sci. 2018 May 3;19(5):1357. doi: 10.3390/ijms19051357 (PMC5983645; doi:10.3390/ijms19051357)
Supplement: Supplementary file 1 [file ijms-19-01357-s001.pdf]

Supplementary Information

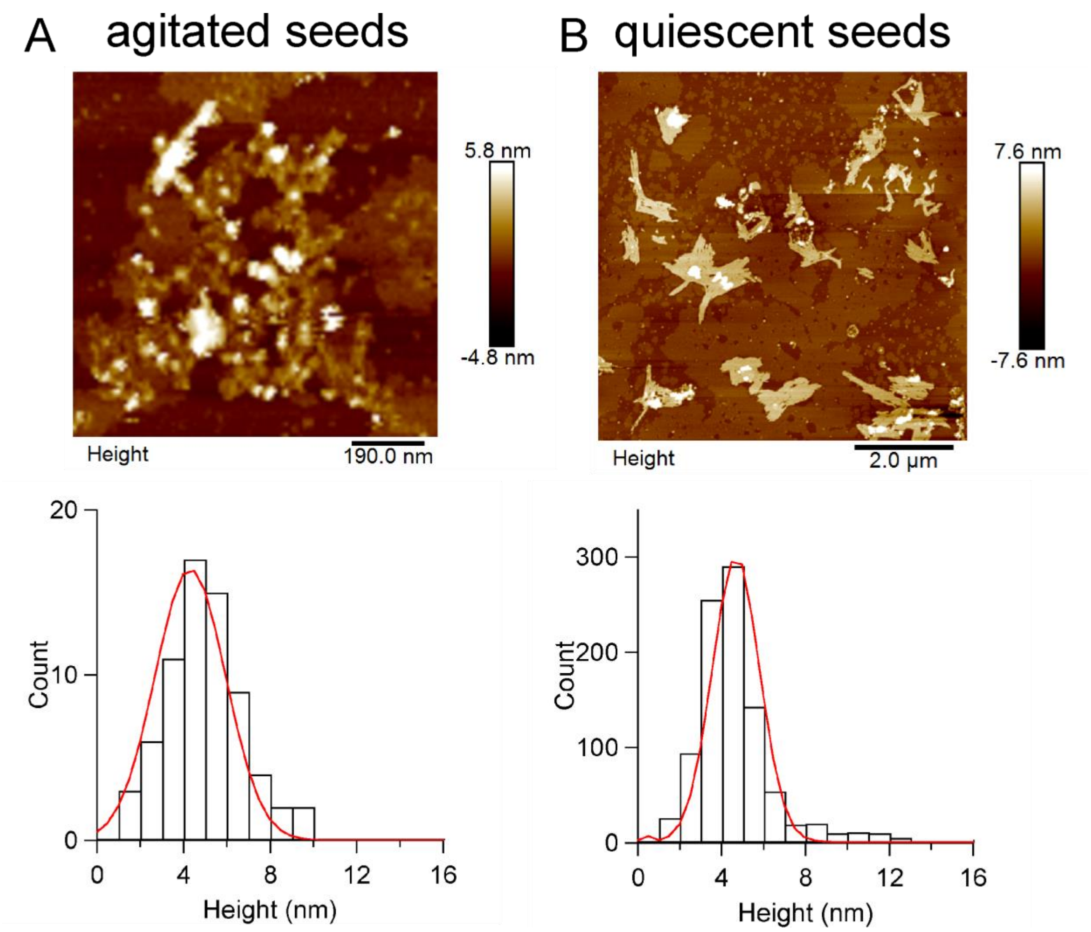

Figure S1: Atomic Force Microscopy height distribution analysis for sonicated fibrils used as seeds. A) Sonicated pre-formed fibrils generated under quiescent conditions shows one predominant species with average height of 4.9 ± 1.5 nm (N = 900). B) Sonicated pre-formed fibrils generated under agitated conditions shows one species with average height 4.5 ± 1.0 nm (N = 70).

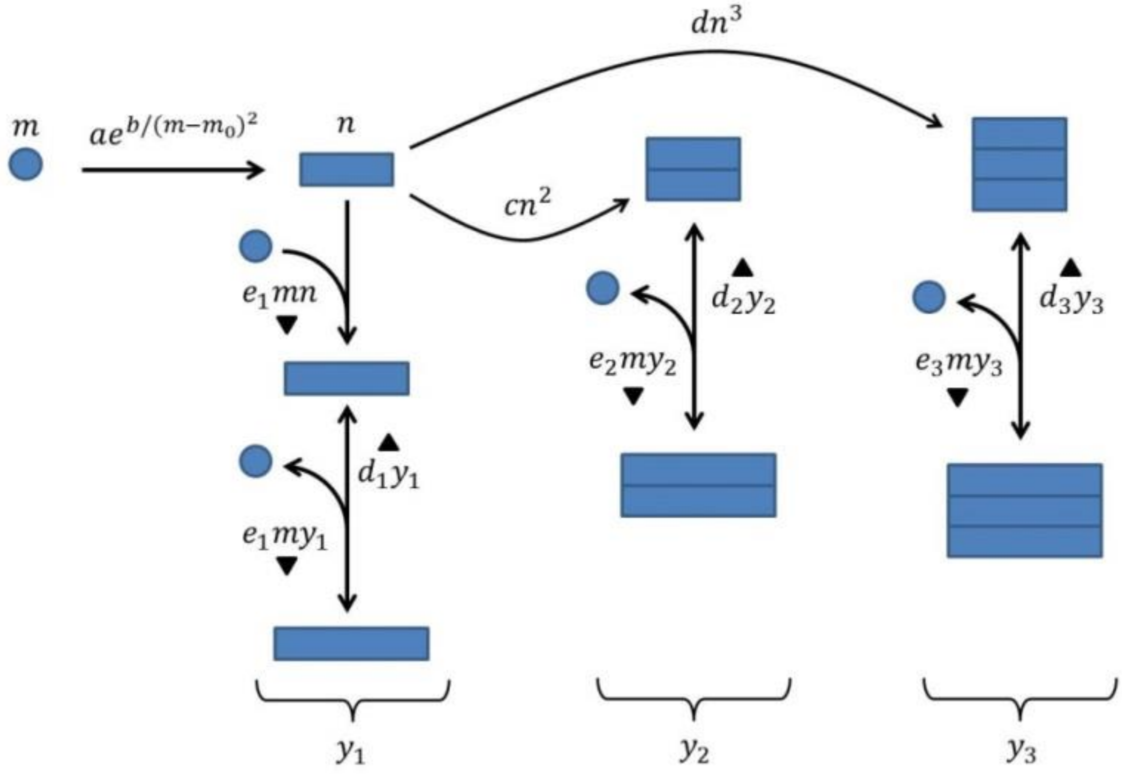

Figure S2: Diagram showing how the model equations relate to fibril assembly and monomer dissociation for the three fibril forms observed. Monomer concentration; *m*, concentration of seeding fibrils; *n*, elongation rate; *e*, dissociation rate; *d*, fibril concentration; *y*, nucleation rate; *a*, total protein concentration; *c*. Triangles indicate the direction which the equations relate to.

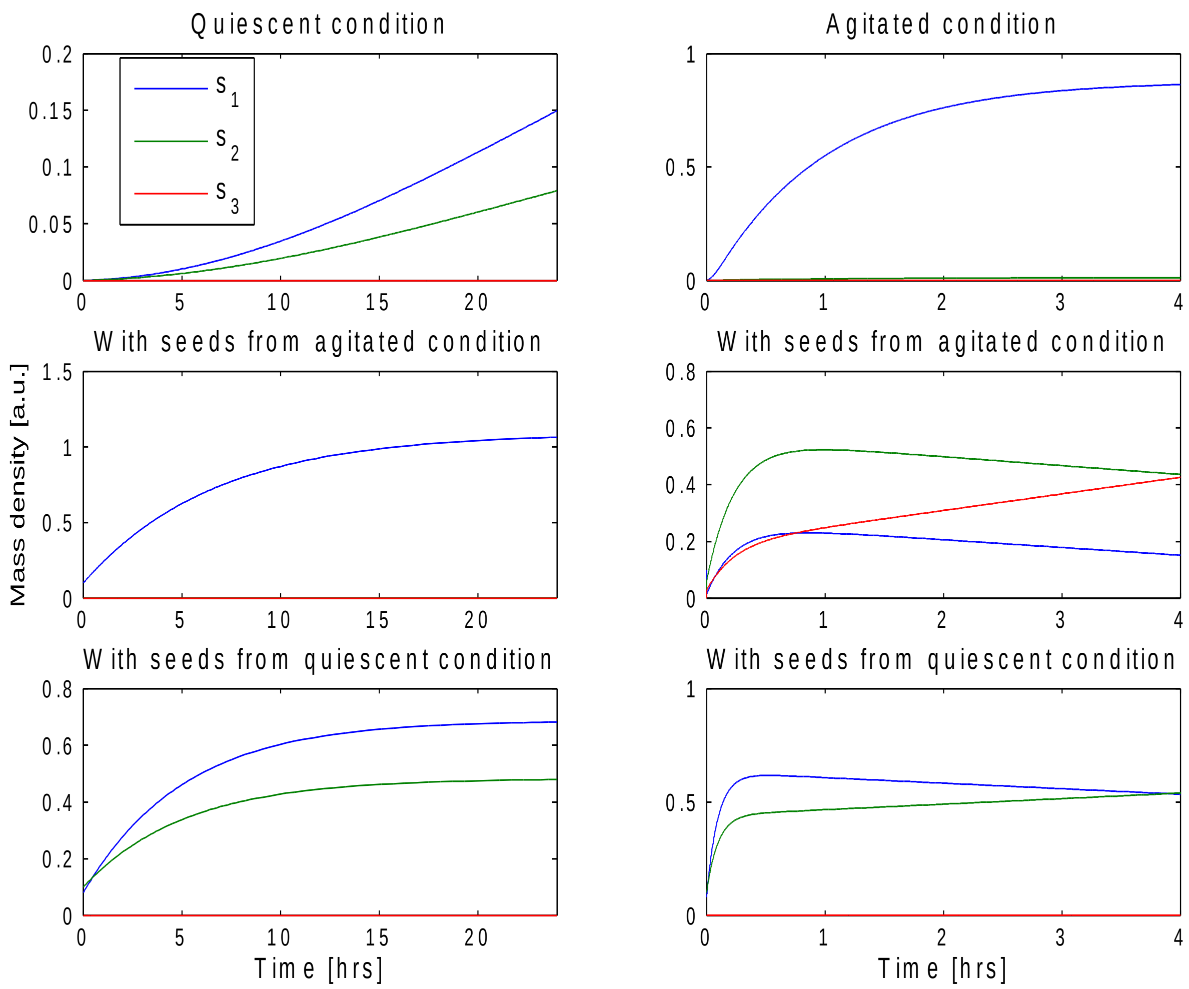

Figure S3: Modelling results based on biophysical model. In this model, nucleation to the narrowest fibrils occur from the monomeric pool. Wider fibrils are subsequently formed by lateral association of the narrowest fibrils. The mass densities of the fibrils of type s_i are denoted by z_i in the model equations.

Table S1: The parameters employed in the simulation with the dimensions of the parameters shown inside the squared brackets. The parameters vary depending on the experimental conditions. The concentration is parameterised by the initial monomer concentration $C \equiv m(t = 0)$.

Parameters	Quiescent	Agitated
$a\ [1/hr]$	0.0015	8
$b\ [C^2]$	1	1
$c\ [1/(C \cdot hr)]$	0	1.6×10^4
$d\ [1/(C^2 \cdot hr)]$	0	1.6×10^6
$e_1\ [1/(C \cdot hr)]$	1.5	800
$e_2\ [1/(C \cdot hr)]$	1.5	800
$e_3\ [1/(C \cdot hr)]$	1.5	800
$f_1\ [1/hr]$	0.15	88
$f_2\ [1/hr]$	0.1	80
$f_3\ [1/hr]$	0	8
N	10	10
$m_0\ [C]$	0.1	0.5

Modelling of fibril nucleation

AFM measurements indicated that there were three species (s_1, s_2, s_3) of fibrils categorised by their heights, which were around 5, 10 and 15 nm respectively (Figure 6). Denoting the monomeric species by m, we considered here the following nucleation schemes:

$$\begin{array}{ccccc} m & \xrightarrow[\alpha_1(m)]{} & s_1 & \xrightarrow[\alpha_2(s_1,\bar{L}_1)]{} & s_2 \\ & & s_1 & \xrightarrow[\alpha_3(s_1,\bar{L}_1)]{} & s_3 \end{array}$$

where α_k are the nucleation rates. Specifically, we assumed that the formation of wider fibrils depended on the existence of the thinnest fibril type s_1, with s_1 being nucleated from monomers alone. Physically this amounted to the assumption that monomers first nucleate to form thin fibrils of width 5 nm (α_1 nucleation). After that, two of these thin fibrils could potentially come together and bind laterally to form a thicker s_2 fibril of width around 10 nm (α_2 nucleation). Similarly, three of them could potentially come together to form a s_3 fibril (α_3 nucleation). In addition, we postulated that nucleation of wider fibrils (α_2 and α_3) depended not only on the concentrations of the constituent fibrils and the experimental condition (whether agitated or quiescent), but also on the lengths of the constituent fibrils ($\bar{L}_1$). This assumption was based on the intuition that it is easier to "braid" a rope out of short fragments of filaments than to do so with long filaments. In other words, we expected that α_2 and α_3 would go down rapidly as s_1 goes down and as $\bar{L}_1$ goes up.

Minimal model

To translate the intuitive picture shown in Figure 6 into a mathematical model, we devised a minimal ordinary differential equation-based model, a set of kinetic equations describing how the concentrations change with time for the four species: monomers, type-1,2,3 fibrils. In particular, within type-1 fibrils, we specifically kept track of the concentration of seeding fibrils, i.e., short type-1 fibrils that can bind together to form type-2 and type-3 fibrils (Figure S2).

In terms of notation, we denote the monomer concentration by m, the concentration of the seeding fibrils by n, the concentration of type-i fibril ($i = 1,2,3$) by y_i, and the monomer concentration assembled in the form of type- i fibril ($i = 1,2,3$) by z_i. The model equations are:

$$\frac{dm}{dt} = -ae^{\frac{b}{(m-m_0)^2}}\Theta(m-m_0) - m(e_1y_1 + e_2y_2 + e_3y_3) + f_1y_1 + f_2y_2 + f_3y_3 \quad [1]$$

$$\frac{dn}{dt} = \frac{ae^{\frac{b}{(m-m_0)^2}}\Theta(m-m_0)}{N} - cn^2 - dn^3 - e_1mn \quad [2]$$

$$\frac{dy_1}{dt} = \frac{ae^{\frac{b}{(m-m_0)^2}}\Theta(m-m_0)}{N} - cn^2 - dn^3 \quad [3]$$

$$\frac{dy_2}{dt} = \frac{cn^2}{2} \quad [4]$$

$$\frac{dy_3}{dt} = \frac{dn^3}{3} \quad [5]$$

$$\frac{dz_1}{dt} = ae^{\frac{b}{(m-m_0)^2}}\Theta(m-m_0) + e_1my_1 - f_1y_1 - N(cn^2 + dn^3) \quad [6]$$

$$\frac{dz_2}{dt} = Ncn^2 + e_2my_2 - f_2y_2 \quad [7]$$

$$\frac{dz_3}{dt} = Ndn^3 + e_3my_3 - f_3y_3 \quad [8]$$

whereby the conservation of protein concentration, $m + z_1 + z_2 + z_3 = C$ where C is the total protein concentration.

In the above equations, we have modelled the nucleation events, fibril elongation and monomer dissociation from the fibrillar ends [1]. We assume that fibrillar breakage is unlikely and thus is negligible with regard to the kinetics.

$m \rightarrow n$ nucleation

The first term on the right hand side of Equation [1] models the nucleation event that produces the seeding fibrils n and $\Theta(.)$ denotes the Heaviside function. To model this nucleation event, we approximate the nucleation rate from the classical theory of nucleation where $m - m_0$ denotes the supersaturation [2]. Note that we have kept the exponential term that varies strongly with m while taken the prefactor to be a factor for simplicity. Microscopically, the nucleation events may result from micelles in the solution [3, 4] or from the interface due to the surface activity of the monomers [5], here we employ this analytical expression phenomenologically.

$n \rightarrow y_i$ nucleation

The formation of type-2 fibril is taken to be induced by the dimerisation of the two seeding fibrils. Mathematically, the rate of this transition is modelled using mass action law: cn^2 (second term on the right hand side of Equation [2]. Similarly, the rate of type-3 fibrils formation is given by dn^3 (third term on the right hand side of Equation [2].

Elongation

The elongation of fibrils are modelled again using mass action law and thus by the terms e_1mn and e_imy_i (second to fourth terms in Equation [1]).

Monomer dissociation from the fibrillar ends

Given the typical multi-filament structure of an amyloid fibril, we assume that monomer detaches from the fibrils primarily from the ends.[1] These effects are modelled by the last three terms in Equation [1]. We also assume that these events are only significant when the monomer pool is sufficiently depleted. As a result, we ignore the disappearance of fibrils due to monomer dissociation in our model equations.

Model parameters

The drastically simplified model we consider here already has 12 free parameters, which are different depending on whether the system is in the quiescent or agitated conditions. In addition, the system's behaviour depends on the initial conditions (seeding or not). Given the complexity of the model, we will focus here purely on capturing the qualitative behaviour of the experimental observations. The model parameters employed are shown in Table S1.

Initial conditions

Besides the model parameters, we also needed to fix the initial conditions of the model equations. In the non-seeding condition, the initial condition was such that all concentrations are zero except m, which was set to be 1. When sonicated seeds incubated in the quiescent condition were added at the beginning, the initial conditions were: $m = 1, y_1 = 0.008, y_2 = 0.005, z_1 = 0.08, z_2 = 0.1$ while all other concentrations are zero. For sonicated seeds incubated in the agitated condition, the initial conditions were $m = 1, n = 0.01, y_1 = 0.01, z_1 = 0.1$ and all other concentrations were again zero.

Relation to experimental findings (Figure S3)

Quiescent condition. Under the quiescent condition (Q), both s_1 and s_2 can be nucleated because upon nucleating s_1, the elongation remains slow such that $\bar{L}_1$ remains small for a long enough time that the α_2 nucleation pathway is not negligible. Hence, nucleation from s_1 to s_2 is possible. This explains why both species are present in the quiescent condition.

Agitated condition. In the agitated condition, nucleation to s_1 is possible but as elongation rate is higher than in the quiescent condition, $\alpha_{2,3}$ are suppressed (because $\bar{L}_1$ becomes high very quickly), and as a result, only s_1 exists in the system.

Sonication. Sonication leads to breakage of fibrils due to the shearing of the fluid imposed by sonication. Here, we assume that for the fibrils formed under the quiescent condition, sonication leads to a reduction of $\bar{L}_1$ and $\bar{L}_2$, while for fibrils formed under the agitated condition, sonication leads to a reduction of $\bar{L}_1$ for the s_1 species, the only species in the system.

Seeding - A+A$_s$. When seeds formed by sonicated fibrils prepared under the agitated condition (A$_s$) are put back with monomers and incubated under the agitated condition, we found that species s_2 and s_3 dominate the system. This reflects the fact that at the beginning of the experiments, there is an abundance of seeds of the form s_1 with small $\bar{L}_1$, both $\alpha_{2,3}$ are thus non-negligible and so some s_1 fibrils are converted to s_2 and s_3 fibrils. However, *de*

novo nucleation from monomers is suppressed because of the rapid elongation rate that leads to a rapid decrease in m.

Seeding - A+Q$_s$. When seeds formed by sonicating fibrils prepared under the quiescent condition (Q_s) are put back with monomers and incubated under the agitated condition, both s_1 and s_2 are present with small $\bar{L}_1$ and $\bar{L}_2$ due to sonication. Compared to the seeds A_s, the seed concentration of type s_1 is less abundant here and so the nucleation pathway to s_3 remains negligible. As a result, only two species (s_1 and s_2) of fibrils exist in the system.

Seeding - Q+A$_s$. Here, we have an abundance of seeds of type s_1 and $\bar{L}_1$ is small at the beginning. However, $s_{2,3}$ are negligible under the quiescent condition, so there are negligible amounts of $s_{2,3}$ fibrils at the beginning. In addition, the large number of seeds also leads to a rapid elongation of s_1 fibrils and the associated depletion of monomers, thus both $\alpha_{2,3}$ nucleation are also negligible. Therefore, only type s_1 fibrils remain in the system.

Seeding - Q+Q$_s$. This is similar to the situation in the scenario A+Q_s, except that the elongation rate is smaller since we are under the quiescent condition, but the conclusion that only s_1 and s_2 are present remain true.

Supplementary References

1. Trigg, B.J., C.F. Lee, D.J. Vaux and L. Jean, The air–water interface determines the outcome of seeding during amyloidogenesis. *Biochem J*, **2013**. 456: p. 67-80, DOI: 10.1042/BJ20130605
2. Barrat, J.L. and J.P. Hansen, *Basic Concepts for Simple and Complex Liquids*. **2003**: Cambridge University Press.
3. Lomakin, A., D.B. Teplow, D.A. Kirschner and G.B. Benedek, Kinetic theory of fibrillogenesis of amyloid β-protein. *Proc Nat Acad Sci*, **1997**. 94: p. 7942-7947.
4. Lee, C.F., Self-assembly of protein amyloids: A competition between amorphous and ordered aggregation. *Physical Review E*, **2009**. 80: p. 031922-031922, DOI: 10.1103/PhysRevE.80.031922
5. Jean, L., C.F. Lee, C. Lee, M. Shaw and D.J. Vaux, Competing discrete interfacial effects are critical for amyloidogenesis. *FASEB J,* **2010**. 24: p. 309-317, DOI: 10.1096/fj.09-137653
